# Clinical Outcome Following Concussion Among College Athletes with a History of Prior Concussion: A Systematic Review

**DOI:** 10.1186/s40798-022-00528-6

**Published:** 2022-10-29

**Authors:** Charles E. Gaudet, Grant L. Iverson, Alicia Kissinger-Knox, Ryan Van Patten, Nathan E. Cook

**Affiliations:** 1 Department of Physical Medicine and Rehabilitation, Harvard Medical School, Boston, MA USA; 2 MassGeneral Hospital for Children Sports Concussion Program, Waltham, MA USA; 3 Department of Physical Medicine and Rehabilitation, Spaulding Rehabilitation Hospital, Charlestown, MA USA; 4 Providence Veterans Administration Medical Center, Providence, RI USA; 5 Department of Psychiatry and Human Behavior, Alpert Medical School of Brown University, Providence, RI USA; 6 Center for Health and Rehabilitation Research, Charlestown, MA 02129 USA

**Keywords:** Concussion, Mild traumatic brain injury, Clinical recovery, Prior concussions, College athletes

## Abstract

**Background:**

There is long-standing interest in, and concern about, whether collegiate athletes with a history of concussion will experience worse clinical outcomes, or prolonged recovery, should they sustain a subsequent concussion.

**Objectives:**

This systematic review examined the association between prior concussion history and clinical outcomes following a subsequent sport-related concussion among college-age student athletes.

**Study Design:**

Systematic review.

**Methods:**

We screened 5,118 abstracts and 619 full-text articles that were appraised to determine whether they met inclusion criteria. We utilized a likelihood heuristic to assess the probability of observing a specific number of statistically significant and nonsignificant studies reporting an association between concussion history and clinical outcomes. We conducted a narrative synthesis of the study findings.

**Results:**

Sixteen studies met the inclusion criteria. Thirteen studies reported the number of participants with a history of prior concussions (≥ 1), which totaled 1690 of 4573 total participants (on average 37.0% of study participants; median = 46.0%, range 5.6–63.8%). On the Newcastle–Ottawa Quality Assessment Scale, the risk of bias ratings ranged from 3 to 9 (mean = 5.4, SD = 1.4). Across all studies, 43.8% (*k* = 7/16) reported at least one statistically significant result among primary analyses showing an association between concussion history and worse clinical outcome. A minority of studies reporting on symptom duration (4/13, 30.8%) and time to return to play (2/7, 28.6%) found an association between concussion history and worse outcome. Studies included in the review reported limited information pertaining to the characteristics of prior concussions, such as presence or duration of loss of consciousness or posttraumatic amnesia, age at first lifetime concussion, time since most recent past concussion, or length of recovery from prior concussions.

**Conclusion:**

The question of whether college athletes with a prior history of concussion have worse clinical outcome from their next sport-related concussion remains unresolved. The published results are mixed and in aggregate show modest evidence for an association. Many studies have small samples, and only three studies were designed specifically to address this research question. Important outcomes, such as time to return to academics, have not been adequately studied. Larger hypothesis-driven studies considering the number of prior concussions (e.g., 3 or more) are needed.

*Trial registration*: PROSPERO CRD42016041479, CRD42019128300.

**Supplementary Information:**

The online version contains supplementary material available at 10.1186/s40798-022-00528-6.

## Key Points

- The association between prior concussion history and clinical outcome following a subsequent sport-related concussion remains uncertain among collegiate athletes.
- The most commonly studied clinical outcomes, symptom duration and time to return to sports, did not show an association with prior concussion history in the majority of studies.
- There are numerous features of prior concussion history, such as the number of prior concussions and corresponding courses of recovery, which have not been sufficiently examined and represent areas for future research.

## Introduction

The most recent Consensus Statement on Concussion in Sport identified obtaining a “detailed concussion history” as a requisite component of both preparticipation and postinjury evaluations [1]. Among collegiate athletes, National Collegiate Athletic Association (NCAA) Injury Surveillance Program data suggest that approximately 9–14% of athletes sustain multiple, or “recurrent,” concussions during their collegiate careers, irrespective of their concussion history prior to enrolling in college [2, 3]. Among NCAA Division I hockey players, players with a prior concussion history were twice as likely to sustain a subsequent concussion [4]. Hence, at minimum, approximately 1 in 10 collegiate athletes will present with a prior history of at least one concussion. Although a history of prior concussion is a well-established risk factor for sustaining additional, future concussions [5–8], the association between prior concussion history and clinical outcome following a subsequent concussion has not been well established, as acknowledged by the consensus statement [1]. Given the unique demands faced by collegiate athletes, such as balancing academic and athletic responsibilities, enhancing clinicians’ ability to accurately identify potential factors associated with prolonged or complicated clinical recovery following concussion is critically important. To this end, characterizing whether or the extent to which concussion history influences clinical recovery from subsequent concussion represents an important undertaking.

In a broad systematic review investigating a wide range of predictors of clinical recovery from sport-related concussion in athlete populations of all ages, of the studies examining concussion history, approximately half (48.8%; 20/41) reported a statistically significant association between concussion history and clinical recovery—such that individuals with a history of prior concussions experience worse clinical outcomes or slower clinical recoveries. The remaining studies (51.2%; 21/41) reported no association between concussion history and clinical outcome [9]. A more recent systematic review examined the association between prior concussion history and clinical recovery following sport-related concussion in children and adolescents. Across the 51 studies reviewed, the majority (37/51; 72.5%) did not find a statistically significant association between lifetime history of concussion and outcome following subsequent sport-related concussion [10]. However, associations between concussion history and clinical outcomes in *collegiate athletes* have not been specifically characterized—hence the need for an updated assessment of the literature.

Evolving methodological advancements in the study of concussion history, such as considering the exact number of prior concussions, rather than studying concussion history as a dichotomous variable (i.e., any prior concussions: yes/no), has begun to yield more nuanced insights. For example, a recent study of collegiate athletes reported that a history of three or more prior concussions was associated with longer clinical recoveries; however, this association was not detected in athletes with only one or two prior concussions [11]. Moreover, concussion history is a complex variable, and its prognostic utility may be reduced if it is measured in a binary fashion (i.e., yes or no). It is conceivable, for example, that the total number of prior concussions might have a stronger association with clinical outcome than a simple binary variable. Additional considerations, such as (1) the age of first concussion; (2) the nature, severity, and recovery times of prior concussions; (3) and time since the most recent prior concussion could conceivably bear on clinical outcomes following a subsequent “index” concussion. Through this systematic review, we will (1) examine associations between concussion history and clinical outcomes following subsequent sport-related concussion among college-age athletes, including results related to the nature and magnitude of effects; (2) assess the methodological quality of the literature and identify gaps in the existing research; and (3) offer recommendations for future research.

## Methods

An original systematic review was prospectively registered with PROSPERO database for systematic reviews (protocol ID: CRD42016041479). A second, updated systematic review was subsequently registered with PROSPERO (protocol ID: CRD42019128300). The current review followed Preferred Reporting Items for Systematic Reviews and Meta-analyses (PRISMA) guidelines [12]. For the purpose of the present review, clinical outcome was broadly defined to include self-reported resolution of concussion-related symptoms, time to return to play and/or resume normal activities, and changes in neuropsychological and/or vestibular functioning.

### Search Strategy

We identified articles by online database searching: PubMed, MEDLINE®, PsycINFO®, CINAHL, Cochrane Library, EMBASE, SPORTDiscus, Scopus, and Web of Science and hand-searching reference lists. Three partially overlapping searches were conducted. The first was from database inception to June of 2016 for a systematic review prepared for the 2016 Concussion in Sport Group conference in Berlin [13]. The second was from January 1, 2016, to February 1, 2019 [14, 15], and the third was from February 1, 2019, to May 15, 2021. Given the frequency with which multiple predictor variables, including concussion history, are included as a demographic variable, secondary predictor, or covariate, we included a broad set of terms to increase the probability of capturing the array of predictors associated with clinical outcome from prior concussion—per the design of the 2016 systematic review. The following terms were used across searches: sport, sports [MeSH]), athletic, athlete, player AND craniocerebral trauma, brain injuries, brain concussion, sports concussion, athletic injuries, mild traumatic brain injury, mTBI, traumatic brain injury, TBI, brain concussion, concussion, multiple concussions, repeated concussion, repetitive concussion, cumulative concussions, concussion history, brain damage, prognosis, outcome, recovery, risk factor, injury incidence, sex differences, gender, genetics, ApoE, BDNF, S100B, GFAP, severity, loss of consciousness, LOC, posttraumatic amnesia, PTA, amnesia, retrograde amnesia, seizure, seizures, learning disorder, ADHD, level of education, migraine, mental health, sleep disorders, medications, cervical injury, vestibular injury, psychological reactions, anxiety, depression, headaches, intractable headaches, magnetic resonance imaging, MRI, computer tomography, and CT.

### Study Selection and Data Extraction

**Fig. 1:**
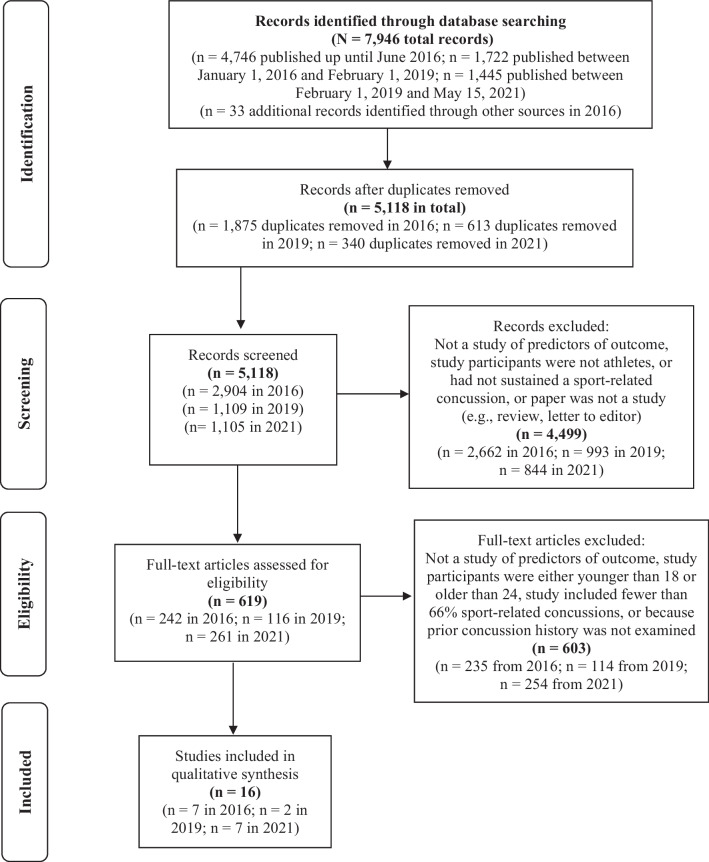
Systematic literature search (PRISMA). Note: There was a mistake in the reporting of the number of records identified in the original search from 2016, as published by Iverson et al. in 2017. The initial search in 2016 yielded 4746 records, as noted in this figure.

The search sequence and selection flow are displayed in Fig. 1. Original studies, including descriptive, correlational, quasi-experimental, and experimental designs, were eligible for inclusion. We excluded case studies and published abstracts. Our primary interest was in selecting studies that reported on collegiate athletes and examined whether a history of prior concussion was associated with outcome from a subsequent sport-related concussion. To this end, several inclusion criteria were applied. We included studies that reported an association between concussion history and outcome in any way (e.g., in text only, in table only, concussion history considered as a demographic variable, secondary outcome, or covariate). Studies reporting only on acute clinical outcome less than 7 days following the index concussion and not reporting clinical recovery were excluded. Regarding the mechanism of concussion, our aim was to focus primarily on sport-related concussion. Hence, at least two-thirds (66%) of the participants in the studies had to present with a sport-related mechanism for the index injury (i.e., the injury for which they were enrolled in the study and their recovery was examined). Studies were also restricted to collegiate athletes, or college-aged individuals. College-age was defined as the lower bound of the standard deviation for the study sample’s mean age being greater than or equal to 18 years, and the upper bound being less than or equal to 24 years. This ensured that at least 84% of the study samples were comprised of individuals between ages 18 and 24.

The authors independently extracted details regarding study design, sample demographics, concussion history coding (e.g., how the study examined or categorized concussion history such as ≥ 1 or more prior concussions vs. 1, 2, or ≥ 3 prior concussions) and features of prior concussions (e.g., age of first lifetime concussion, time since last concussion, mechanism of last concussion, clinical characteristics of prior concussions [loss of consciousness, retrograde amnesia, posttraumatic amnesia], recovery times). The authors also extracted results of statistical significance testing (e.g., results in which *p* < 0.05) and/or effect size metrics evaluating the association between concussion history and outcome from the index concussion, if available from the information reported in the study. The first author (CEG) extracted information from each article and combinations of co-authors served as second raters (NEC, RVP, AKK). In instances in which there was uncertainty regarding a study’s reporting, a third author resolved discrepancies.

### Risk of Bias Assessment and Level of Evidence Ratings

Risk of bias was assessed using the Newcastle–Ottawa Quality Assessment Scale (NOS) [16]. The NOS is used for observational studies, including cohort and case–control studies. It is comprised of nine items distributed among the following categories: Sample Selection, Comparability between Research Groups, and Outcome (for Cohort studies) or Exposure (for Case Control studies). Higher ratings are indicative of higher-quality studies and lower risk of bias. For the purpose of the present review, NOS criteria were appraised in relation to each study’s assessment of the association between concussion history and clinical outcome following a subsequent concussion. Concussion history was considered as the “exposure” and clinical outcome (e.g., symptom duration, time to return to play) as the “outcome.” Consequently, based on the original study design (which in most cases was not primarily designed to assess concussion history), a study might have earned credit on one of the NOS parameters. However, when we examined the study in relation to its ability to determine whether prior concussion history is associated with worse outcome from a subsequent concussion, the study might not have earned credit on that parameter. Level of evidence for each article was rated by the first author (CEG) and a second rater (NEC, RVP, or AKK) using the Oxford Classification for Evidence-Based Medicine. The first author (CEG) also rated risk of bias for each article along with a second rater (NEC, RVP, or AKK). A third reviewer resolved rating discrepancies.

### Likelihood Ratio Estimation

Given the range of clinical outcomes reported across studies and lack of uniformity in, or absence of, effect size metrics, the data were not amenable to commonly used meta-analytic procedures. We therefore utilized a likelihood heuristic to assess the probability of observing a specific number of statistically significant and nonsignificant studies reporting an association between concussion history and clinical outcomes. The likelihood heuristic yields a likelihood ratio (LR) that signals the weight of the evidence in support of the null hypothesis (i.e., concussion history *is not* associated with clinical outcomes following subsequent concussion) versus the alternative hypothesis (i.e., concussion history *is* associated with worse clinical outcomes) [17]. The likelihood heuristic has been used to characterize associations between sport-related concussion and clinical outcomes in prior systematic reviews [15, 18].

To compute the LRs, an a priori alpha (e.g., 0.05) and statistical power value (e.g., 0.80) are selected. Next, the number of studies that reported a statistically significant result (*k*) for various outcomes (e.g., symptom duration, time to return to play) are summed and input as a proportion of the overall number of studies reporting on a specific outcome (*n*). To compute LRs, we used a freely available LR calculator (https://lakens.shinyapps.io/likelihood/) that has been developed to assist in synthesizing results from multiple studies [17]. Given the variance in design, quality, and sample sizes among the studies included in the review, we conducted two pairs of analyses in which we varied our parameters to illustrate the range of LRs resulting from optimal and suboptimal study designs. For the first analysis, we selected an alpha level of 0.05 and power level of 0.80, which would reflect a near-optimal study design. For the second analysis, we selected an alpha level of 0.25 and power level of 0.45, which is more likely to capture shortcomings inherent to several of the study designs included in the present review. In accordance with established guidelines, LR estimates were characterized as follows: LRs greater than 8 and 32 are considered benchmarks of moderate and strong evidence, respectively [17, 19].

## Results

A total of 5118 abstracts were screened, and 619 full-text articles were appraised to determine whether they met inclusion criteria (see Fig. 1). Of those, 16 studies met inclusion criteria. Additional file 1: Table S1 provides detailed information regarding the included studies. There were 4841 participants who sustained an index concussion across the 15 studies that reported the number of participants. The study that did not report a total number of participants reported that 1670 concussions were sustained during the study period [3]. Thirteen (81.3%) of the studies were published since 2012, and 12 (75.0%) were published since 2016. Of the seven studies that reported a mean age for the total sample, the median age of the means was 20.3 years. Three studies reported the age range comprising the entire sample; the lowest bound was age 17 and the highest bound was age 27. The studies included a median of 30.3% women (range 0–55.6%). Thirteen studies reported the number of participants with a history of prior concussions (≥ 1), which totaled 1690 of 4573 total participants (on average 37.0% of study participants; median = 46.0%, range 5.6–63.8%). Fifteen studies reported only on collegiate athletes (both varsity and club level) seeking care through their institution. One study reported on participants who sought care at a specialty concussion clinic, and 92.2% of the concussions included in this study were sport-related [20]. The majority of the studies included in this review assessed symptom duration (*k* = 13) or time to return to sports (*k* = 7); additional outcomes included vestibular functioning (*k* = 2), time to return to academics (*k* = 1), cognitive functioning (*k* = 1), and psychiatric problems (*k* = 1) [Table 1]. Of note, seven studies reported multiple outcomes; hence, the numbers above sum to greater than 16.

**Table 1:** Summary of the study outcomes (studies listed alphabetically)

First author (year)	Total N	Prior concussion n		Concussion history coding	Prior concussion during study	Concussion history is study focus	Symptom duration			Time to return to play			Other outcomes		
							Significant			Significant			Significant		
							Findings			Findings			Findings		
		f	%				Yes	No	Direction	Yes	No	Direction	Yes	No	Direction
Asken et al. [26]	97	48	49.5%	0, 1, ≥ 2	No	No					X	–			
Bretzin et al. [33]	1,974	1,030	52.2%	0, 1, 2, ≥ 3	NR	No		X	–		X	–	X^a^	X	Worse and Null
Bruce and Echemendia [22]	57	30	52.6%	≥ 1 Prior	No	**Yes**	X		Worse						
Churchill et al. [27]	33	19	57.6%	≥ 1 Prior	No	No		X	–						
Gallagher et al. [28]	90	34	37.8%	≥ 1 Prior	No	No		X	–						
Guskiewicz et al. [23]	184	66^b^	NR	0, 1, 2, ≥ 3	Yes	**Yes**	X		Worse						
Howell et al. [35]	94	60	63.8%	≥ 1 Prior	NR	No								X^c^	^–^
Lempke et al. [29]	187	86	46.0%	≥ 1 Prior	No	No	± X ±	X	Worse and Null	± X ±	X	Worse and Null			
Meehan et al. [20]	64	30	46.9%	≥ 1 Prior	No	No		X	–						
Mihalik et al. [24]	45	19	37.8%	0, 1, ≥ 2	Yes & No	No		X	–	X		Worse			
Pattinson et al. [32]	127	54	42.5%	0, 1, 2, ≥ 3	NR	No		X	–						
Putukian et al. [30]	138	64	46.4%	0, 1, ≥ 2	No	No		X	–		X	–			
Slobounov et al. [21]	160	9	5.6%	0, 1	Yes	**Yes**		X	–		X	–	X^c^	X^d^	Worse and Null
Vargas et al. [31]	84	NR	NR	Continuous	No	No								X^e^	–
Wasserman et al. [3]	1,670^f^	151^f^	9.0%	≥ 1 Prior	Yes	No	X		Worse	X		Worse			
Zuckerman et al. [2]	1,507	210	13.9%	≥ 1 Prior	Yes	No	X		Worse						

^a^Time to return to academics; prior concussion associated with longer time to return in men but not in women

^b^This figure reflects the total number of prior concussions, not the number of participants with prior concussions

^c^Vestibular functioning

^d^Cognitive functioning

^e^Depressive symptoms

^f^This study reported number of concussions and not the number of participants who sustained a concussion

± X ± This study reported that concussion history was *not* associated with symptom duration or time to return to play. However, among athletes who sustained concussions during away competitions (versus at home), concussion history was associated with longer symptom duration and time to return to play. Therefore, for the purpose of this review, in tabulating the number of “positive” studies, we did not count this one as a positive study (e.g., in Table 3). “Prior Concussion During Study” refers to whether concussion history was assessed in the study based on concussions sustained before the study (“No”), during the study (“Yes”), both before and during the study (“Yes & No”), or was not reported (“NR”)

### Level of Evidence and Risk of Bias

NOS ratings are shown in Table 2. Of the included studies, 15 were classified as cohort designs and one was classified as a case control study. The mean Centre for Evidence-Based Medicine (CEBM) level of evidence was 3.1 (SD = 0.5). One study was rated as a 2 (i.e., inception cohort), 12 studies were rated as a 3 (i.e., cohort studies), and 3 studies were rated as a 4 (i.e., case–control or lower-quality prognostic cohort studies). On the NOS, ratings ranged from 3 to 9 (mean = 5.4, SD = 1.4). The majority of cohort studies were deemed representative of the average collegiate sport-related concussion (*k* = 11). Only four studies received at least one credit for comparability. This was primarily attributable to study design and lack of statistical control for covariates (e.g., gender, mental health status) that may be associated with clinical outcomes. Most studies allowed sufficient follow-up time for clinical outcomes to occur, although they varied in their methodology for assessment of outcomes (i.e., record linkage vs. self-report) and reporting on the number of participants lost to follow-up.

**Table 2:** The Newcastle–Ottawa Quality Assessment Scale scores and the level of evidence of the included studies (listed alphabetically)

First author (year)	Design^a^	Newcastle–Ottawa Scale^b^				CEBM
		Selection (0–4)	Comparability (0–2)	Outcome/exposure (0–3)	Total credits	Level of evidence (1–5)
Asken et al. [26]	Cohort	★★☆★	☆☆	★★☆	5	3
Bretzin et al. [33]	Cohort	★★☆★	★☆	★★☆	6	3
Bruce and Echemendia [22]	Cohort	★★☆★	☆☆	☆★☆	4	4
Churchill et al. [27]	Cohort	★★☆★	★☆	☆★☆	5	3
Gallagher et al. [28]	Cohort	★★★★	☆☆	★★☆	6	3
Guskiewicz et al. [23]	Cohort	☆★☆★	☆☆	★★★	5	3
Howell et al. [35]	Cohort	★★☆★	☆☆	★★☆	5	3
Lempke et al. [29]	Cohort	★★★★	★★	★★★	9	4
Meehan et al. [20]	Cohort	☆★☆★	☆☆	☆★★	4	3
Mihalik et al. [24]	Cohort	☆★☆★	☆☆	★★☆	4	3
Pattinson et al. [32]	Cohort	★★☆★	☆☆	★★★	6	3
Putukian et al. [30]	Cohort	★★☆★	☆☆	☆★★	5	3
Slobounov et al. [21]	Cohort	☆★★★	☆☆	★★★	6	2
Vargas et al. [31]	Case–Control	★★★☆	☆☆	☆☆☆	3	4
Wasserman et al. [3]	Cohort	★★★☆	☆☆	★★★	6	3
Zuckerman et al. [2]	Cohort	★★★☆	★★	☆★★	7	3

CEBM = Centre for Evidence-Based Medicine

^a^For the NOS, we determined the study design in reference to the determination of whether prior concussion history is a predictor of worse clinical outcome

^b^When completing the NOS, we rated the study in relation to whether prior concussion history is a predictor of worse clinical recovery from concussion. Thus, in certain circumstances, the original study design might have earned credit on one of the NOS parameters based on the predictor variables of interest in that study (which were not prior concussion history). However, when we conceptualized the study with prior concussion history as the primary variable of interest, it did not receive credit on that same parameter (e.g., because appropriate covariates of prior concussion history were not examined)

### Limited Information Relating to Prior Concussions

Reporting on features related to prior concussion history and the period for when prior concussion history was measured (i.e., before or during the study period) is presented in Additional file 1: Table S2. Nine of the 16 studies (56.3%) coded and analyzed concussion history dichotomously (i.e., 0, ≥ 1 prior) although three of these studies reported greater detail surrounding the number of prior concussions (i.e., 0, 1, ≥ 2) in their demographic sections. Prior concussion mechanism was not reported in the majority of studies; only 25% of studies (*k* = 4) reported on the mechanism of the previous concussion. Age at first concussion was only reported in one study, and this appeared attributable to the study’s inception cohort design [21]. The time interval since the most recent concussion preceding the index concussion was reported in 25.0% of studies (*k* = 4); in these studies, the interval ranged from within the past year to a median of 2 years. The majority of studies (68.8%, *k* = 11) reported the method for determining prior concussion history; six relied on self-report, two relied on athletic trainer report, two evaluated medical records, and one involved a clinical assessment (attributable to its inception cohort design [21]). Regarding injury severity characteristics of prior concussions, aside from the study with an inception cohort design [21], none of the studies reported on clinical characteristics such as loss of consciousness, retrograde amnesia, or anterograde amnesia. Similarly, only one study reported recovery times associated with prior concussions (e.g., > 3 days, > 1 week, > 28 days), attributable to its inception cohort design [21].

Only 3 of the 16 studies (18.8%) were specifically designed to examine the relation between prior concussion history and outcome following subsequent concussion [21–23]. The association between prior concussion history and clinical outcome varied, with 2 studies reporting on shorter postinjury intervals (i.e., 7 days) and detecting an association between prior concussion history and symptom duration, such that a greater proportion of athletes with prior concussions reported symptoms during this time interval [22, 23]. In contrast, one study did not find an association between prior concussion history and time to return to sport in collegiate rugby players at a slightly longer postinjury interval (10 days) [21]. Notably, all of these studies were conducted over 15 years ago.

Additionally, of the 13 studies that reported on whether prior concussion history referred to concussions sustained prior to the study period, during the study period, or included both, only 5 studies (38.5%) examined prior concussions sustained during the study period [3, 21, 23–25]. Interestingly, each of these 5 studies (100%) reported at least one worse outcome associated with prior concussions sustained during the study period; of the 9 outcomes examined (e.g., symptom duration, time to return to play), worse outcomes were reported for 5 of them (55.6%; see Table 1). In contrast, of the 8 studies (61.5%) for which prior concussions referred to injuries only sustained prior to the study period [20, 22, 26–31], only 2 studies (25%) reported an association between prior concussion history and worse outcome [22, 29].

### Association Between Prior Concussion and Clinical Outcome from Subsequent Concussion

When considering all studies, under the assumption of 80% power and 5% alpha or optimal study design, LR estimates indicate strong evidence of an association between prior concussion history and worse outcome as 7 of the 16 studies (43.8%) found an association (see Table 3). Similarly, there appears to be strong evidence of an association when examining the studies with the lowest risk of bias (*k* = 4/7 studies reporting a statistically significant association with worse outcome). However, changing the parameters relating to power and alpha to mirror what might be more realistic study design conditions dramatically reduces confidence in this association. Moreover, when we consider the outcomes that were most often studied, such as duration of symptoms (*k* = 4/13 studies showing an association) or time to return to play (*k* = 2/7 studies showing an association), likelihood ratios yielded the opposite conclusion, that is, evidence in support of the null hypothesis (i.e., no association).

**Table 3:** Likelihood ratio estimates in support of the null hypothesis (no effect of prior concussion on clinical outcomes)

	Total studies	Studies reporting both “positive” results *and* null results		
		Number of “Positive” studies	Likelihood ratio	
			80% Power, 5% alpha	45% Power, 25% alpha
All studies	16	7	218.07+	3.76+
Symptom duration	13	4	18.78−	1.55−
Return to play	7	2	9.45−	1.46−
Lowest risk for bias^a^	7	4	611.5+	4.14+

“Positive” refers to a statistically significant result (i.e., < 0.05). Studies that reported *both* statistically significant results and null results were classified as reporting “positive” results suggestive of an association between prior concussion history and clinical outcome (with the exception of Lempke et al. [29] as noted in Table 1). + (positive) Higher likelihood ratios (LR) indicate increased likelihood in favor of the alternative hypothesis (i.e., prior concussion history *is associated with* clinical outcomes following subsequent concussion); − (negative) Higher likelihood ratios (LR) indicate increased likelihood in favor of the null hypothesis (i.e., prior concussion history *is not associated* with clinical outcomes following subsequent concussion). Magnitude of LRs was characterized as follows: LR = 1 = no effect, LR between 1 and 8 = weak, LR greater than 8 and less than 32 = moderate, LR greater than 32 = strong. ^a^Greater than 6 credits on the Newcastle–Ottawa Scale

*Symptom Duration*. As can be seen in Table 4, studies assessing symptom duration varied in the length of recovery time measured and analyzed. At earlier postinjury intervals (i.e., ≤ 7 days), two of three studies reported a significant association between concussion history and symptom duration [22, 23]. In a study of NCAA football players, length of symptom recovery was divided into three categories (rapid: < 1 day, gradual: 1–7 days, and prolonged: > 7 days). These symptom duration groups were compared relative to the number of prior concussions (0, 1, 2, ≥ 3) and revealed significant differences between groups (Fisher’s exact test, *p* = 0.03) [23]. Similarly, a smaller scale study of 57 NCAA athletes examined the association between concussion history and symptom duration lasting beyond 7 days. In this study, a greater proportion of athletes with a history of prior concussion (32%) endorsed cognitive/balance symptoms at 1 week postinjury compared to those with no concussion history (8%) [22]. In contrast, in a study of 45 collegiate athletes whose symptoms resolved within one week (Mdn = 6.1 days), concussion history and symptom duration were not associated [24].

**Table 4:** Symptom duration outcome intervals and prior concussion history

Study	Outcome coding	Result		*p*	Effect size
Mihalik et al. [24]	NR (~ 6 days)	Odds ratio reflects time to symptom resolution in those with prior concussion history relative to those without prior concussion. Median symptom resolution time for the overall sample was 6.1 days		NR	1.13 [0.62, 2.04]^a^
Bruce and Echemendia [22]	7 days	Percentage endorsing cognitive/balance symptoms Prior concussion group: 32% No prior concussion group: 8%		**< 0.05**	NR
Guskiewicz et al. [23]	< 1 day; 1–7 days; > 7 days	Length of symptom recovery 0 Prior concussions: < 1 day: 30.3%; 1–7 days: 62.3%; > 7 days: 7.4% 1 Prior concussion: < 1 day: 39.0%; 1–7 days: 46.3%; > 7 days: 14.6% 2 Prior concussions: < 1 day: 33.3%; 1–7 days: 46.7%; > 7 days: 20.0% ≥ 3 Prior concussions: < 1 day: 0.0%; 1–7 days: 70.0%; > 7 days: 30.0%		**0.03**	NR
Slobounov et al. [21]	10 days	All participants were clinically asymptomatic on day 10 of testing		NR	NR
Pattinson et al. [32]	14 days	The number of prior concussions did not differ between those who experienced symptom resolution at < 14 days and ≥ 14 days		0.13	NR
Churchill et al. [27]	RTP; 28 days	No difference between those with and without concussion history at any of the time points		≥ 0.21	NR
	Post-RTP; 1 year post-RTP				
Meehan et al. [20]	28 days	Symptom Resolution (days) ≤ 28 days: 66.6% of participants with prior SRCs; 50.0% of those without SRC > 28 days: 33.3% of participants with prior SRCs; 50.0% of those without SRC		0.61	NR
Wasserman et al. [3]	28 days	14.6% of athletes with recurrent SRCs symptoms lasted > 28 days 5.4% of athletes with no recurrent SRCs took symptoms lasted > 28 days		**< 0.001**	NR
Zuckerman et al. [2]	28 days	13.3% of athletes with recurrent SRCs symptoms lasted > 28 days 6.5% of athletes with no recurrent SRCs took symptoms lasted > 28 days		**< 0.001**	2.08 [1.28–3.36]
Bretzin et al. [33]	Continuous (until recovery)	Median Days [IQR]	Median Days [IQR]	0.104	NR
		Men:	Women:		
		0 prior concussions: 7 [4, 15]	0 prior concussions: 9 [5, 17]		
		1 prior concussion: 9 [4, 14]	1 prior concussion: 9 [5, 22]		
		2 prior concussions: 10 [5, 24]	2 prior concussions: 9 [4, 20]		
		≥ 3 prior concussions: 10 [4, 33]	≥ 3 prior concussions: 13 [8, 38]		
Gallagher et al. [28]	Continuous (until recovery)	No relationship between concussion history and time since injury to physician clearance date (assumes symptom resolution)		> 0.05	NR
Lempke et al. [29]^b^	Continuous (until recovery)	≥ 1 Prior concussion not associated with days to symptom recovery		0.139	0.6 [− 0.2, 1.4]^c^
Putukian et al. [30]	Continuous (until recovery)	Days until symptom free was not significantly associated with concussion history		> 0.05	NR

Primary study results shown in the table. In some instances, a subset of the study population showed a conflicting association between prior concussion and clinical outcome

RTP = Return to play; SRC = sport-related concussion

^a^Negative binomial regression coefficient

^b^A subset of the sample (athletes who sustained concussions during away competitions) did show a significant association between concussion history and symptom duration

^c^Multivariable regression measure estimate [95% confidence interval]

The majority of studies examining symptom duration did so at 10 or more days postinjury or in a continuous manner. A study of NCAA athletes that assessed symptom duration as a continuous variable investigated the effect of on-field heat index and altitude on clinical recovery from concussion. This study had mixed results. In a multivariable model that included the full study sample, concussion history was not associated with symptom duration (*p* = 0.139). However, concussion history was associated with symptom duration in the subset of the sample who sustained concussions during away competitions—the reasons for this finding were unclear but may have been related to differences in the on-field heat index and altitude between home and away venues. Moreover, the magnitude of this difference was small and likely not clinically meaningful (as having at least one prior concussion corresponded to an additional 0.99 days in symptom duration, *p* = 0.05) [29]. Aside from this isolated result from a select subset of athletes from a larger sample, most studies did not report statistically significant results. In a prospective study of men and women participating in collegiate rugby with no prior concussion history, athletes who sustained a second concussion during the study period (*N* = 9; hence, prior concussion history) were all clinically asymptomatic by 10 days postinjury [21]. Similarly, in a study of 127 NCAA athletes across multiple sports, prior concussion history was not associated with time to symptom resolution (i.e., < 14 days vs. ≥ 14 days) [32]. A large-scale study of collegiate athletes from Ivy League schools did not show an association between number of prior concussions and symptom duration in men (*p* = 0.104) or women (*p* = 0.560) [33]. Four additional studies of collegiate athletes also did not show an association between concussion history and symptom duration [20, 27, 28, 30].

Two studies reported statistically significant results at longer postinjury intervals (i.e., 28 days). In a study examining NCAA Injury Surveillance Program data from the 2009–2010 to 2013–2014 seasons, a greater proportion of athletes who sustained recurrent concussions showed longer symptom resolution times (i.e., > 28 days) than those who sustained a single concussion (14.6% vs. 5.4%, *p* < 0.001) [3]. Another study examining the NCAA’s Injury Surveillance Program data from the 2009–2010 to 2014–2015 seasons revealed that athletes with recurrent concussion were more likely to experience persistent symptoms (i.e., > 28 days) in both univariable (13.3% vs. 6.5%, OR = 2.22 [95% CI, 1.41–3.50], *p* < 0.001) and multivariable analyses (OR = 2.08 [95% CI, 1.28–3.36]) [2]. Interestingly, a study, conducted at a concussion specialty clinic, examined the ongoing presence of concussion symptoms at 28 days postinjury and reported no differences between those with and without concussion histories; however, a higher percentage of participants with prior concussions reported ongoing symptoms (33.3% of participants with prior sport-related concussions vs. 40.0% of participants with no prior sport-related concussions) relative to the NCAA Injury Surveillance Program studies [20].

*Return to Play.* Seven studies reported on time to return to play, and 71.4% (*k* = 5) did not report an association between concussion history and time to return to play. In a study of 97 NCAA athletes, 48 of whom had a history of prior concussions, there was no difference in the proportion of athletes with a prolonged time to return to play based on concussion history (≥ 8 days; *p* = 0.56) [26]. Similarly, a study of 138 collegiate athletes, 64 of whom had prior concussions, did not report an association between concussion history and time to return to play [30]. In a large-scale study of Ivy League athletes, time to return to play, measured as both time until initial clearance to gradually resume activities and time until full participation clearance, did not show an association with number of prior concussions in either men (*p* = 0.174, *p* = 0.327, respectively) or women (*p* = 0.367, *p* = 0.575, respectively)[33]. Additionally, time to return to play did not differ between those with and without a history of prior concussion in a sample of university-level athletes in Canada [34].

In contrast, two of seven studies (28.6%) reported an association between concussion history and longer times to return to play. Data from the NCAA’s Injury Surveillance Program indicated that a higher proportion of athletes with recurrent concussions took longer to return to play (> 28 days) than those who did not have recurrent concussions (21.2% vs. 7.7%; *p* < 0.001) [3]. Similarly, in a study of 45 NCAA football players, those with a prior concussion history displayed 86% longer return to play times than those with no prior concussion history (OR = 1.86 [95% CI, 1.06–3.28]), although, interestingly, there was no difference in symptom duration time. The study’s authors speculated that this discrepancy may have been attributable to more conservative management of players with a concussion history by healthcare personnel [24]. In the previously discussed study that examined the effect of an on-field heat index and altitude [29], concussion history was not associated with return to play times in a multivariable model (*p* = 0.091). However, similar to the relation between concussion history and symptom duration, in the subset of athletes who sustained concussions during away competitions, a history of one or more concussions was associated with an additional 2.88 days in time to return to play (*p* = 0.008).

*Time to Return to Academics.* Only one study examined time to return to academics and reported mixed results. Among collegiate men (*n* = 1209), Kaplan–Meier survival analyses revealed an association between number of prior concussions and time to return to academics (*p* = 0.022); however, a statistically significant association between time to return to academics and number of prior concussions was not reported among collegiate women (n = 765; *p* = 0.900) [33].

*Cognitive Functioning.* One study reported on the association between concussion history and cognitive functioning. This study was comprised of men and women who participated in collegiate rugby and reported that players who sustained multiple concussions during the study period and those who did not performed at baseline levels on cognitive testing by 10 days postinjury. Notably, cognitive functioning was a secondary outcome in the study, and information describing cognitive measures and scores was not presented [21].

*Vestibular Functioning.* Two studies reported on the association between concussion history and vestibular functioning. In a study of men and women participating in collegiate rugby, relative to players with no prior concussions, those with a prior concussion history showed differences in vestibular functioning as measured by virtual reality motion testing (*p* < 0.001) at least 30 days following injury [21]. In contrast, in a study assessing dual-task gait recovery among Division 1 NCAA athletes, concussion history was not associated with single-task gait recovery (HR = 1.033, *p* = 0.92) or dual-task gait recovery (HR = 1.301, *p* = 0.50) at less than 7 days postinjury, approximately 1.5 months postinjury, or approximately 3.5 months postinjury [35].

*Psychiatric Functioning.* One study assessed the association between the number of prior concussions and self-reported depressive symptoms following an index concussion both within 48 h and 5 days (range 1–41 days) postinjury. This study did not report a significant association between concussion history and depressive symptomatology (*r* = 0.16, *p* = 0.15) [31].

## Discussion

This systematic review identified 16 studies that examined the association between prior concussion history and clinical outcomes following subsequent sport-related concussion in college-aged athletes. The mean CEBM level of evidence rating was 3.1 and NOS risk of bias rating was 5.4. The literature is mixed with 43.8% of the studies reporting a statistically significant association among their primary analyses between prior concussion history and worse clinical outcome. The majority of studies reported on either symptom duration (*k* = 13) and/or time to return to play (*k* = 7). Additional clinical outcomes included time to return to academics (*k* = 1), cognitive functioning (*k* = 1), vestibular functioning (*k* = 2), and psychiatric functioning (*k* = 1). Concussion history was primarily reported as a demographic variable or covariate and was rarely examined as a primary predictor or focus of the study. Only *three studies were explicitly designed to examine the association* between concussion history and outcome. Effect sizes characterizing the magnitude of association between concussion history and clinical outcomes were not commonly reported nor able to be calculated and meta-analyzed. Descriptive information relating to the nature and clinical characteristics of prior concussions was severely limited. Overall, based on the number and quality of studies reporting statistically significant results, there is only modest evidence in support of an association between a history of concussion and worse clinical outcome following a subsequent sport-related concussion in college student athletes. At present, the extant literature, considered in toto, remains inconclusive. There are numerous qualifications and caveats regarding the generalizability of the literature.

This review is comprised of studies that reported both significant and null results for the majority of clinical outcomes assessed. Of the studies that assessed symptom duration, results were mixed, and a minority (30.8%; *k* = 4) reported at least one statistically significant association between concussion history and symptom duration in their primary analyses. Two of the studies that reported significant results reported on a brief postinjury outcome interval (i.e., symptoms persisting more than 7 days) [22, 23]. Two other studies appeared to have examined overlapping samples from the NCAA Injury Surveillance Program [2, 3]. The remaining studies (*k* = 9) reported no association between prior concussion history and symptom duration. Likelihood ratios provided evidence in support of the null hypothesis (i.e., no association between concussion history and symptom duration; Table 3). Taken together, studies assessing the association between prior concussion and symptom duration following subsequent sport-related concussion did not yield evidence that allows for a clear inference regarding the presence of an association. Among the studies reporting on additional clinical outcome, such as return to academics, cognitive functioning, psychiatric functioning, and vestibular functioning, there were too few studies to draw any conclusions.

Of the studies that reported on time to return to play, results also were mixed and a minority (28.6%; *k* = 2) reported at least one statistically significant result showing an association between concussion history and greater time to return to play. Notably, one of those studies reported no association between symptom duration and concussion history, leading the authors to speculate that clinicians at their study sites managed athletes differently based on their reported concussion history and thus held them out of sports longer despite a similar symptom duration to those athletes without a concussion history [24]. Research examining concussion management trends among NCAA football players suggests that concussion management practices have become more conservative over time. For example, among samples of NCAA football players, between 1999 and 2001 the median time to return to play was only 3.0 days, whereas between 2014 and 2017 the median time to return to play was 12.2 days [36]. In sum, although a few studies report longer time to return to play among athletes with prior concussions, it is unclear the extent to which evolving concussion management protocols or clinician decisions/management practices may have contributed to the few observed differences.

Several studies not included in this review that reported on prior concussion history and clinical outcomes bear mentioning. These studies were excluded due to a sizeable proportion of high-school aged students included in their sample and the authors not reporting results of interest separately for college-aged athletes. Two studies conducted in specialty concussion clinics did not report an association between number of prior concussions and symptom duration [37, 38]. Similarly, a study consisting of adolescents and young adults (age range 13–27 years) who sustained sport-related and non-sport-related concussion did not detect an association between the number of prior concussions and acute symptom severity, recovery time, or the proportion of participants recovered within 28 days [39]. Additionally, symptom duration in high school and collegiate athletes was not associated with either recurrent concussion (i.e., same season repeat concussion; OR = 1.14, 95% CI = 0.57, 2.31, *p* = 0.71) or number of prior concussions (*p* = 0.44) [40]. Another large-scale study comprised of 8,905 collegiate and 7513 high school athletes reported no association between concussion history and symptom duration, cognitive functioning, and vestibular functioning by three days postinjury [41].

### Limitations

Our systematic review has several limitations. The most important limitation is that most studies were not designed specifically to examine the association between a personal history of one or more prior concussions and outcome from the athletes’ current concussion. Instead, these studies treated concussion history more like a demographic variable or covariate, not a primary hypothesis-associated variable of interest. Statistically, several of the studies were likely underpowered to detect group differences on the basis of concussion history due to small sample and cell sizes. Moreover, reporting of effect sizes was limited—thereby precluding inferences regarding the overall magnitude of effects on clinical outcomes. This limitation also restricted our ability to synthesize data using traditional meta-analytic methods; however, we offset this limitation to some extent through the use of a likelihood heuristic.

The review was limited to English language studies; hence, a language-of-publication bias may be present. Relatedly, only published studies were included in the review, raising the risk of publication bias with possible underrepresentation of studies that did not reveal statistically significant associations (the so-called file-drawer problem). In regard to the nature of the topic, many of the studies relied on self-report of prior concussion history or did not report the method for gathering this information. Consequently, the reliability of the reported number of prior concussions and proportions of samples with prior concussions are uncertain. Relatedly, there is the potential for varying definitions of concussion being used across studies. The majority of studies (*k* = 9) analyzed the association between concussion history and clinical outcomes with concussion history coded as a binary variable (i.e., ≥ 1 prior, yes/no). Although such procedures may be necessitated by the nature of available data, analyzing concussion history as a dichotomous predictor of clinical outcomes does not allow for assessment of differences between individuals with only one prior concussion versus those who may have sustained three or more, for example. Lastly, we do not know how prior concussions were clinically assessed and managed. Studies included in the review were published between 2004 and 2021. Concussion management practices have evolved significantly over this 17-year period. Hence, for example, associations between concussion history and clinical outcome for concussions sustained in 2005 may differ from prior concussions sustained in 2020 as a function of differences in management practices.

### Future Directions

More focused, adequately powered, hypothesis-driven research on this topic is needed as prior concussion history will continue to be a variable of interest in research assessing both short- and long-term clinical outcomes following sport-related concussion. As will be described below, addressing methodological gaps identified in this review represents an initial pathway toward advancing our knowledge and understanding of the potential effects of multiple concussions. To this end, we believe there are seven important considerations.

First, we should aim to measure and report on the number of prior concussions more precisely. Obtaining continuous (i.e., total number) versus binary (i.e., ≥ 1 prior concussion, yes/no) estimates allows for more detailed and granular analyses. For example, results of a recent study suggest that among former collegiate football players, with no professional football exposure, a history of three or more prior concussions, but not a prior history of only one or two concussions, was associated with worse health outcomes (e.g., physical pain, depressive symptomatology, general health) 15 years after the conclusion of their athletic careers [42]. This apparent threshold of three or more prior concussions, versus only one or two, has been cited as a prognostic indicator for worse clinical outcomes in other studies as well [11, 23, 42, 43]. Additionally, a study conducted across 3 university-affiliated specialty clinics, comprised of 270 adolescents, reported that a history of 3 or more prior concussions increased risk for experiencing persistent postconcussion symptoms. Of note, the subsample of adolescents with 3 or more prior concussions was small (*n* = 15). Approximately 67% of adolescents with 3 or more prior concussions, 38% with 2 or more prior concussions, 38% with 1 or more prior concussions, and 32% with no prior concussions experienced persistent symptoms. Additional factors known to contribute to clinical recovery were not controlled for, such as gender, symptom burden, and psychiatric status [44]. However, in the absence of more robust data collection procedures, and large sample sizes, knowledge related to whether, or the extent to which, the *number* of prior concussions is associated with clinical outcomes and whether there is a certain threshold (e.g., 3 or more) that increases risk for worse outcomes, will remain uncertain.

Second, clinical features associated with prior concussions may be of value in better understanding the potential association between prior concussion history and clinical outcome from subsequent concussion and should be reported in future research. The time interval between prior concussion and the index concussion is likely a topic of importance [45]. Results of the present review indicated that prior concussions sustained during the study period, as opposed to before the study period, and thus closer in time to the index concussion were more frequently associated with worse clinical outcomes following a subsequent concussion. The time course of recovery from prior concussions may be of considerable importance in mediating an association between concussion history and clinical outcome. For example, there might be a subgroup of athletes who have experienced swift recoveries from prior concussions and are thus more resilient and optimistic, and less prone to health-related anxiety that may interfere with recovery from a subsequent concussion. In contrast, there may be a subgroup of athletes that has experienced a challenging and prolonged recovery from a prior concussion and subsequently is more susceptible to factors that might prolong recovery from a subsequent concussion. In a large study of children and adolescents, concussion history, per se, was not a strong predictor of having symptoms lasting more than a month—but having a prior concussion with symptom duration greater than 7 days was an independent risk factor for having prolonged symptoms with their current concussion [46]. Additional data that might have prognostic value include the age at time of first concussion [47, 48], prior concussion injury mechanisms [49], and acute symptom severity of prior concussions [50].

Third, half of the studies included in this review had cell sizes of those with prior concussions of 50 or fewer. Larger, hypothesis-driven studies are needed. That said, future researchers conducting extremely large studies, with sample sizes in the thousands or tens of thousands, are encouraged to interpret their statistically significant findings in close association with the effect sizes, because very small magnitude differences that are statistically significant might have limited practical or clinical importance.

Fourth, and related to clinical outcomes, we must strive for inclusion of social determinants of health in study designs, particularly in accounting for concussion history. Recent research has revealed that although approximately equivalent percentages of White and Black athletes reported a history of concussion nondisclosure, reasons for nondisclosure differed by race [51]. Moreover, social determinants warrant consideration in the assessment of clinical outcomes following concussion, because factors such as health insurance (public vs. private), commonly cited as a proxy for socioeconomic status, have been associated with time to return to school [52]. Additional research has used ZIP code and health insurance provider as proximal indicators of socioeconomic status and has yielded mixed results in regard to time to recover from concussion among adolescents [53, 54]

Fifth, examination of the association between prior concussions and a broader range of clinical outcomes is warranted. Only three studies in this review were primarily designed to assess whether having prior concussions was associated with worse clinical outcome from a subsequent concussion; the remaining studies typically included prior injury history as a demographic variable or covariate. Additionally, numerous studies examined symptom duration and time to return to play; however, few studies examined time to return to academics, cognitive functioning, psychiatric functioning, or vestibular functioning. As it relates to collegiate athletes, and college students in general, time to return to academics (without accommodations) is among the most critical outcomes and is likely to have a significant impact on well-being. Recommendations and guidelines for return to academics following concussion tailored to college students are emerging [55, 56]. Developing a more advanced understanding of academic outcomes among college students who have returned to school following concussion may support the development and refinement of such recommendations. Whether a history of prior concussions has a mediating or moderating effect on college students’ time to return to school following concussion will be an important consideration in this pursuit.

Sixth, as it relates to college-age populations, we should expand beyond collegiate athletes to better understand how concussion history interacts with outcomes in college students who are not participating in sports. Results from a recent large university cohort study revealed that approximately 44% of students who were not athletes, and who sustained an index concussion while in college, reported a personal history of prior concussion. Index concussions reported in this study resulted from a variety of mechanisms, such as falls, motor vehicle accidents, objects falling on their head, and physical altercations [57]. Given that this population likely does not have equivalent access to athletic training and rehabilitative services that collegiate athletes receive, better understanding of their clinical outcomes might allow for more effective provision of healthcare services.

Seventh, we should aim to better understand mechanisms underlying an association between prior concussion history and worse clinical outcomes. Neuroimaging research has reported differences in cingulate cerebral blood flow between athletes with and without a history of concussion at 1 year following return to play from concussion. Such findings were present in the absence of between group differences on cognitive screening and in time to return to play [58].

## Conclusion

The question of whether college athletes with a prior history of concussion have, on average, worse clinical outcome from their next concussion remains unresolved. The studies published to date report mixed findings, and in aggregate show only modest evidence for an association. Many studies to date are small, and only three focused specifically on this topic. Moreover, early studies defined prolonged recovery as greater than 7–10 days, whereas in the past few years that time period is considered subacute and it is common for athletes to be going through a gradual process of recovery and return to play during that time period. Therefore, longer time to return to play among some athletes with prior concussions may be attributable to changing protocols towards more conservative medical management, over the past 15 years, rather than reflecting a true deleterious effect of prior concussions on recovery time from a subsequent injury. This will require careful study to disentangle. Important clinical outcomes such as time to return to academics, cognitive functioning, psychiatric functioning, and vestibular functioning have not been adequately studied. Larger hypothesis-driven studies that consider the number of prior concussions (e.g., 3 or more) are needed to better understand whether those with multiple prior concussions are more likely to experience prolonged clinical recovery, and why.

## Supplementary Information

**Additional file 1: Table S1**. Study Information (listed alphabetically). **Table S2**. Prior Concussion Details (studies listed alphabetically)

## Data Availability

All data and material reported in this systematic review are from peer‐reviewed publications.

## Abbreviations

HR: Hazard ratio
LR: Likelihood ratio
NOS: Newcastle–Ottawa Quality Assessment Scale
OR: Odds ratio
PRISMA: Preferred Reporting Items for Systematic Reviews and Meta-Analyses
