# Supplementary material for: Clinical Outcome Following Concussion Among College Athletes with a History of Prior Concussion: A Systematic Review
Source: Sports Med Open. 2022 Oct 29;8:134. doi: 10.1186/s40798-022-00528-6 (PMC9617993; doi:10.1186/s40798-022-00528-6)
Supplement: Supplementary file 1 — Additional file 1: Table S1. Study Information (listed alphabetically). Table S2. Prior Concussion Details (studies listed alphabetically) [file 40798_2022_528_MOESM1_ESM.docx]

**Online Supplementary Material**

**Title:** Clinical Outcome Following Concussion Among College Athletes with a History of Prior Concussion: A Systematic Review

**Authors:** Charles E. Gaudet, Ph.D., Grant L. Iverson, Ph.D., Alicia Kissinger-Knox, Psy.D., Ryan Van Patten, Ph.D., & Nathan E. Cook, Ph.D.

| **Contents** | **Page** |
| --- | --- |
| Supplementary Table 1. Study Information (listed alphabetically) | 2 |
| Supplementary Table 2. Prior Concussion Details (studies listed alphabetically) | 5 |
| References: Studies Included in the Systematic Review | 7 |

**Supplementary Table 1.** **Study Information (listed alphabetically)**

|  |  |  |  |  |  | **Characteristics of Most Recent (Index) Concussion** | | | | | |  |
| --- | --- | --- | --- | --- | --- | --- | --- | --- | --- | --- | --- | --- |
| **Study #** | **First Author (Year)**  **PMID** | **Total *N*** | **% Women** | **Age in Years**  **Mean (SD), range)** | **Setting** | **Time Since Injury** | **% LOC** | **%**  **AOC** | **%**  **PTA** | **% Retro** | **Mechanism** | **Length of follow-up** |
| 1 | Asken et al. (2016) [1] 27111584 | N=97 | 22.7% | 20.4 (1.3), NR | Collegiate Athletics | NR | NR | NR | NR | NR | Sport | Until recovery (up to 67 days) |
|  | Design: Retrospective cross-sectional study  Outcome: Time to return to play (≤7days=normal recovery; ≥8 days=prolonged recovery) | | | | | | | | | | | |
|  | Findings: *Concussion history was not associated with prolonged recovery* (χ^2^ = 1.15, *p* = .56). | | | | | | | | | | | |
| 2 | Bretzin et al. (2021) [2] 33626145 | N=1,974 | 38.7% | Men: 19.96 (1.3), NR  Women: 19.57 (1.2), NR | Collegiate Athletics | NR | NR | NR | NR | NR | Sport | Until recovery |
|  | Design: Descriptive epidemiologic (injury surveillance study)  Outcome: Duration of symptoms, time to return to play (initial clearance & full participation), and time to return to academics | | | | | | | | | | | |
|  | Findings: *Number of prior concussions was not associated with symptom duration or time to return to play; however, number of prior concussions was associated with time to return to academics among men, but not women.* | | | | | | | | | | | |
| 3 | Bruce & Echemendia (2004) [3] 15505180 | N=57 | 0% | Prior Concussion Group: 20.2 (1.6), NR  No Prior Concussion Group: 20.2 (1.6), NR  Control Group: 19.8 (1.2), NR | Collegiate Athletics | 2 hours | NR | NR | NR | NR | Sport | 1 week |
|  | Design: Prospective cohort study  Outcome: Duration of symptoms | | | | | | | | | | | |
|  | Findings: *Concussion history was associated with fewer reported symptoms at 2 hours post injury (p < 0.05) and no differences at 48 hours post injury. At 1 week post injury, concussion history was associated with greater levels of cognitive/balance symptoms than those with no prior concussion history.* | | | | | | | | | | | |
| 4 | Churchill et al. (2020) [4]  32388345 | N=33 | 51.5% | 20.3 (2.0), NR | Collegiate Athletics | 1-7 days | NR | NR | NR | NR | Sport | 1 year post return to play |
|  | Design: Prospective cohort study  Outcome: Duration of symptoms and time to return to play | | | | | | | | | | | |
|  | Findings: *Concussion history was not associated with symptom severity at any time point (acute phase, medical clearance to return to play, 1 month post return to play, and 1 year post return to play) or time to return to play.* | | | | | | | | | | | |
| 5 | Gallagher et al. (2018) [5] 29336208 | N=90 | 55.6% | Men: 19.8 (1.2), NR  Women: 19.6 (1.1), NR | Collegiate Athletics | Within 3 days | 2.2% | NR | 11.1%^b^ | | Sport | Until recovery |
|  | Design: Retrospective cohort study  Outcome: Duration of symptoms | | | | | | | | | | | |
|  | Findings: *Concussion history was not associated with symptom duration (p > .05).* | | | | | | | | | | | |
| 6 | Guskiewicz et al. (2003) [6] 14625331 | N=184 | 0% | NR | Collegiate Football | 3 hours | 6.3% | NR | 24.1%^b^ | | Football | Until recovery |
|  | Design: Prospective cohort study  Outcome: Duration of symptoms (rapid [< 1 day], gradual [1-7 days], and prolonged [>7 days]) | | | | | | | | | | | |
|  | Findings: *Number of prior concussions was associated with symptom duration* (Fisher’s exact test, *p* = .03). | | | | | | | | | | | |
| 7 | Howell et al. (2020) [7] 31985574 | N=94 | 50.0% | Men: 20.3 (1.3), NR  Women: 20.1 (1.3), NR | Collegiate Athletics | Within 7 days, Mdn=3, range 1-7 | 4.3% | NR | NR | NR | Sport | Up to 209 days |
|  | Design: Prospective cohort study  Outcome: Height-adjusted gait velocity recovery | | | | | | | | | | | |
|  | Findings: *Concussion history was not associated with single-task gait recovery (HR = 1.033, p = 0.92) or dual-task gait recovery (HR = 1.301, p = 0.50).* | | | | | | | | | | | |
| 8 | Lempke et al. (2021) [8] 33332015 | N=187 | 29.4% | 19.7 (1.4), NR | Collegiate Athletics | <48 hours | 9% | NR | 14% | NR | Sport | Until recovery |
|  | Design: Retrospective cohort study  Outcome: Duration of symptoms and time to return to play | | | | | | | | | | | |
|  | Findings: *Concussion history was not associated with symptom duration or time to return to play. However, among athletes who sustained concussions during away competitions, concussion history was associated with longer symptom duration and time to return to play.* | | | | | | | | | | | |
| 9 | Meehan et al. (2016) [9] 26718812 | N=64 | 46.7% | 21 (2), 18-27 | Specialty Concussion Clinic | Within 21 days; 11.2 (5.2) days | 20.3% | NR | 23.4% | NR | 92.2% Sport | Up to 80 days |
|  | Design: Prospective cohort study  Outcome: Duration of symptoms (≤28 days vs. > 28 days) | | | | | | | | | | | |
|  | Findings: *Concussion history (sport, non-sport, and undiagnosed) was not associated with symptom duration.* | | | | | | | | | | | |
| 10 | Mihalik et al. (2020) [10] 33136240 | N=45 | NR | NR | Collegiate Football | Within 6 hours | NR | NR | NR | NR | Football | Until return to play |
|  | Design: Prospective cohort study  Outcome: Duration of symptoms and time to return to play | | | | | | | | | | |  |
|  | Findings: *Concussion history was associated with time to return to play (Negative binomial coefficient = 1.86 [95% CI, 1.06-3.28]), but not symptom duration (Negative binomial coefficient = 1.13 [95% CI, 0.62-2.04]).* | | | | | | | | | | |  |
| 11 | Pattinson et al. (2020) [11] 32852552 | N=127 | 23.6% | 18.9 (1.3), 17-23 | Collegiate Athletics | Within 21 hours of injury | 5.5% | NR | 18.9% | NR | Sport | Until recovery |
|  | Design: Prospective diagnostic study  Outcome: Symptom duration (dichotomized as <14 days vs. ≥14 days) | | | | | | | | | | | |
|  | Findings: *Prior concussion history did not show a statistically significant association with symptom duration.* | | | | | | | | | | | |
| 12 | Putukian et al. (2021) [12] 30540572 | N=138 | 24.6% | 20.4 (1.41), NR | Collegiate Athletics | 0.83 (1.79), range 0-13 days | NR | NR | NR | NR | Sport | Until recovery; up to 150 days |
|  | Design: Prospective cohort study  Outcome: Symptom duration and time to return to play | | | | | | | | | | | |
|  | Findings: *Prior concussion history was not associated with symptom duration or time to return to play.* | | | | | | | | | | | |
| 13 | Slobounov et al. (2007) [13] 17762746 | N=160 | 47.5% | Men: 21.0  Women: 21.4  Sample Range: 18-25 | Collegiate Rugby | 10, 17, & 30 days | NR | NR | NR | NR | Rugby | 30 days |
|  | Design: Prospective cohort study  Outcome: Symptom duration, cognitive function, and rate of recovery of visual-kinesthetic integration | | | | | | | | | | | |
|  | Findings: *Prior concussion history was not associated with return to sport* *(clinically asymptomatic by day 10 post injury, as determined by symptom report and cognitive testing). Rate of recovery of visual-kinesthetic integration was slower in those with prior concussions.* | | | | | | | | | | | |
| 14 | Vargas et al. (2015) [14] 25643158 | N=84 | 22.6% | 18.4 (0.8), NR | Collegiate Athletics | 6.6 (8.7) days | NR | NR | NR | NR | Sport | Up to 41 days |
|  | Design: Case-control study  Outcome: Depressive symptoms | | | | | | | | | | | |
|  | Findings: *Number of prior concussions was not associated with depressive symptoms (r = 0.16, p = 0.15).* | | | | | | | | | | | |
| 15 | Wasserman et al. (2016) [15]  26546304 | N=1,670^c^ | NR | NR | Collegiate Athletics | NR | 5.6% | 32.3% | 13.9% | 9.8% | Sport | Not specified, at least 4 weeks |
|  | Design: Retrospective cohort (injury surveillance study)  Outcome: Duration of symptoms and time to return to play | | | | | | | | | | | |
|  | Findings: *Recurrent concussion was associated with both longer time to symptom resolution (14.6% vs. 5.4%, p < .001) and time to return to play (21.2% vs. 7.7%, p < .001).* | | | | | | | | | | | |
| 16 | Zuckerman et al. (2016) [16]  27032916 | N=1,507 | 31.2% | NR | Collegiate Athletics | NR | 5.2% | 32.6% | 12.9% | 8.8% | Sport | Not specified, at least 4 weeks |
|  | Design: Retrospective cohort (injury surveillance study)  Outcome: Duration of symptoms | | | | | | | | | | | |
|  | Findings: *Recurrent concussion was associated with persistent symptoms* *in both univariable (13.3% vs. 6.5%, OR = 2.22 [95% CI, 1.41-3.50], p < .001) and multivariable analyses (OR = 2.08 [95% CI, 1.28-3.36]).* | | | | | | | | | | | |

NR = Not reported; Y/N=yes/no; Md=median; IQR=interquartile range; ^a^This study included 2,905 college football players; however, only 184 players sustained incident concussions for a total of 196 concussions during the study period.; ^b^ In this study, “amnesia” is defined as anterograde alone and anterograde plus retrograde; ^c^ This study reported the number of concussions observed during the study period, not the number of participants who sustained concussions.

**Supplementary Table 2. Prior Concussion Details (studies listed alphabetically)**

| **First Author (Year)**  **PMID** | **How prior concussion was coded, analyzed** | **Prior concussion reference period** | **Number of Prior Concussions** | **Mechanisms of Prior Concussions** | **Age at First Concussion** | **Time Since Most Recent Prior Concussion** | **Method of Determining Prior Concussions** | **Injury Severity Characteristics of Prior Concussions** | **Number of Prior Concussion with Symptoms Lasting Greater Than:** | | |
| --- | --- | --- | --- | --- | --- | --- | --- | --- | --- | --- | --- |
|  |  |  |  |  |  |  |  |  | **3 Days** | **1 week** | **28 Days** |
| Asken et al. (2016) 27111584 | ≥1 prior, Y/N | Before Study | 0: 47 (49.5%)  1: 36 (37.9%)  ≥2: 12 (12.6%) | NR | NR | NR | Self-Report | NR | NR | NR | NR |
| Bretzin et al. (2021) 33626145 | 0, 1, 2, ≥3 | NR | Men:  0: 562 (46.5%)  1: 354 (29.3%)  2: 175 (14.5%)  >3: 118 (9.7%)  Women:  0: 382 (49.9%)  1: 211 (27.6%)  2: 92 (12.0%)  >3: 80 (10.5%) | NR | NR | NR | NR | NR | NR | NR | NR |
| Bruce & Echemendia (2004)  15505180 | ≥1 prior, Y/N | Before Study | 0: 27 (47.4%)  1: 17 (29.8%)  ≥2: 13 (22.8%) | NR | NR | >6 months | Self-Report | NR | NR | NR | NR |
| Churchill et al. (2020)  32388345 | ≥1 prior, Y/N | Before Study | 0: 14 (42.4%)  >1: 19 (57.6%) | NR | NR | Mdn=24 months, IQR=[10, 69] | NR | NR | NR | NR | NR |
| Gallagher et al. (2018)  29336208 | NR | Before Study | 0: 46 (51.1%)  1: 23 (25.6%)  2-3: 11 (12.2%) | NR | NR | NR | College Medical Record | NR | NR | NR | NR |
| Guskiewicz et al. (2003)  14625331 | 0, 1, 2, ≥3 | During Study | 0: 122 (3.7%)  1: 41 (5.4%)  2: 15 (10.5%)  ≥3: 10 (12.7%) | NR | NR | NR | Self-Report | NR | NR | NR | NR |
| Howell et al. (2020) 31985574 | ≥1 prior, Y/N | NR | 0: 34 (36.2%)  >1: 60 (63.8%) | NR | NR | >6 months | NR | NR | NR | NR | NR |
| Lempke et al. (2021) 33332015 | NR | Before Study | 0: 101 (54.0%)  >1: 86 (46.0%) | NR | NR | NR | Medical Records | NR | NR | NR | NR |
| Meehan et al. (2016) 26718812 | ≥1 prior, Y/N | Before Study | NR  Prior SRC:  27 (46.9%)  Prior non-SRC: 10 (16.9%)  Undiagnosed: 15 (27.8%) | Sport & Non-Sport | NR | NR | Self-Report | NR | NR | NR | NR |
| Mihalik et al. (2020) 33136240 | ≥1 prior, Y/N | Before & During Study | 0: 25 (56.8%)  1: 17 (38.6%)  ≥2: 2 (4.6%) | NR | NR | NR | NR | NR | NR | NR | NR |
| Pattinson et al. (2020) 32852552 | 0, 1, 2, ≥3 | NR | 0: 71 (55.9%)  1: 43 (33.9%)  2: 9 (7.1%)  ≥3: 2 (1.8%) | NR | NR | NR | NR | NR | NR | NR | NR |
| Putukian et al. (2021) 30540572 | NR  0, 1, >2 | Before Study | 0: 74 (53.6%)  1: 41 (29.7%)  ≥2: 23 (16.7%) | NR | NR | NR | Self-Report | NR | NR | NR | NR |
| Slobounov et al. (2007) 17762746 | 0, 1 | During Study | 0: 29 (76.3%)  1: 9 (23.7%) | Rugby | 18-25 | Within 1 year | Clinical Assessment | GCS=13-15 | NR | NR | 0 |
| Vargas et al. (2015) 25643158 | NR | Before Study | Concussion Grp: M=0.92, SD=1.8, range 0-15  Controls: M=0.57, SD=0.9, range 0-3 | NR | NR | NR | Self-Report | NR | NR | NR | NR |
| Wasserman et al. (2016) 26546304 | ≥1 prior, Y/N | During Study | NR | Sport | NR | NR | Athletic Trainer Report | NR | NR | NR | NR |
| Zuckerman et al. (2016) 27032916 | ≥1 prior, Y/N | During Study | NR | Sport | NR | NR | Athletic Trainer Report | NR | NR | NR | NR |

NR = Not reported; Y/N=yes/no; M=mean; SD=standard deviation; Md=median; IQR=interquartile range; SRC=sport-related concussion

**References: Studies Included in the Systematic Review**

1. Asken BM, McCrea MA, Clugston JR, Snyder AR, Houck ZM, Bauer RM. "Playing Through It": Delayed Reporting and Removal From Athletic Activity After Concussion Predicts Prolonged Recovery. J Athl Train. 2016 Apr;51(4):329-35.

2. Bretzin AC, Esopenki C, D'Alonzo B, Wiebe DJ. Clinical recovery timelines following sport-related concussion in men's and women's collegiate sports. Journal of Athletic Training. 2021:1-7.

3. Bruce JM, Echemendia RJ. Concussion history predicts self-reported symptoms before and following a concussive event. Neurology. 2004 Oct 26;63(8):1516-8.

4. Churchill NW, Hutchison MG, Graham SJ, Schweizer TA. Neurometabolites and sport-related concussion: From acute injury to one year after medical clearance. NeuroImage: Clinical. 2020;27:102258.

5. Gallagher V, Kramer N, Abbott K, Alexander J, Breiter H, Herrold A, et al. The Effects of Sex Differences and Hormonal Contraception on Outcomes after Collegiate Sports-Related Concussion. J Neurotrauma. 2018 Jun 1;35(11):1242-7.

6. Guskiewicz KM, McCrea M, Marshall SW, Cantu RC, Randolph C, Barr W, et al. Cumulative effects associated with recurrent concussion in collegiate football players: the NCAA Concussion Study. JAMA. 2003 Nov 19;290(19):2549-55.

7. Howell DR, Oldham J, Lanois C, Koerte I, Lin AP, Berkstresser B, et al. Dual-Task Gait Recovery after Concussion among Female and Male Collegiate Athletes. Med Sci Sports Exerc. 2020 May;52(5):1015-21.

8. Lempke LB, Lynall RC, Le RK, McCrea M, McAllister T, Schmidt JD, et al. The Effects of On-Field Heat Index and Altitude on Concussion Assessments and Recovery Among NCAA Athletes. Sports Medicine. 2021;51:825-35.

9. Meehan WP, 3rd, O'Brien MJ, Geminiani E, Mannix R. Initial symptom burden predicts duration of symptoms after concussion. J Sci Med Sport. 2016 Sep;19(9):722-5.

10. Mihalik JP, Chandran A, Powell JR, Roby PR, Guskiewicz KM, Stemper BD, et al. Do Head Injury Biomechanics Predict Concussion Clinical Recovery in College American Football Players? Annals of Biomedical Engineering; 2020. p. 2555-65.

11. Pattinson CL, Meier TB, Guedes VA, Lai C, Devoto C, Haight T, et al. Plasma Biomarker Concentrations Associated with Return to Sport following Sport-Related Concussion in Collegiate Athletes-A Concussion Assessment, Research, and Education (CARE) Consortium Study. JAMA Network Open. 2020;3:1-11.

12. Putukian M, Riegler K, Amalfe S, Bruce J, Echemendia R. Preinjury and Postinjury Factors That Predict Sports-Related Concussion and Clinical Recovery Time. Clinical journal of sport medicine : official journal of the Canadian Academy of Sport Medicine. 2021;31:15-22.

13. Slobounov S, Slobounov E, Sebastianelli W, Cao C, Newell K. Differential rate of recovery in athletes after first and second concussion episodes. Neurosurgery. 2007 Aug;61(2):338-44.

14. Vargas G, Rabinowitz A, Meyer J, Arnett PA. Predictors and prevalence of postconcussion depression symptoms in collegiate athletes. J Athl Train. 2015 Mar;50(3):250-5.

15. Wasserman EB, Kerr ZY, Zuckerman SL, Covassin T. Epidemiology of Sports-Related Concussions in National Collegiate Athletic Association Athletes From 2009-2010 to 2013-2014: Symptom Prevalence, Symptom Resolution Time, and Return-to-Play Time. Am J Sports Med. 2016 Jan;44(1):226-33.

16. Zuckerman SL, Yengo-Kahn AM, Buckley TA, Solomon GS, Sills AK, Kerr ZY. Predictors of postconcussion syndrome in collegiate student-athletes. Neurosurg Focus. 2016 Apr;40(4):E13.
